# Evaluation of Tear Production as Measured by Schirmer Test I in Dogs after Acepromazine and Acepromazine–Methadone Premedication

**DOI:** 10.3390/ani11113015

**Published:** 2021-10-20

**Authors:** Claudia Giannetto, Francesco Macrì, Annastella Falcone, Elisabetta Giudice, Rosalia Crupi, Luca Cicero, Giovanni Cassata, Francesco Staffieri, Simona Di Pietro

**Affiliations:** 1 Department of Veterinary Sciences, University of Messina, 98168 Messina, Italy; claudia.giannetto1@unime.it (C.G.); francesco.macri@unime.it (F.M.); annastella.falcone@unime.it (A.F.); egiudice@unime.it (E.G.); rcrupi@unime.it (R.C.); dipietros@unime.it (S.D.P.); 2 Instituto Zooprofilattico Sperimentale della Sicilia “A. Mirri”, Via Gino Marinuzzi 3, 90100 Palermo, Italy; giovanni.cassata@izssicilia.it; 3 Department of Emergency and Organ Transplantation, University of Bari, 70124 Bari, Italy; francesco.staffieri@uniba.it

**Keywords:** acepromazine, dog, exposure keratitis, methadone, tear production

## Abstract

**Simple Summary:**

Different sedatives and anesthetic drugs have been reported to cause adverse ocular side effects, such as an exposure keratopathy due to loss of eyelid reflex, lagophthalmos, reduced stability of the tear film and decreased basal tear production. In the present study, the effects of two sedation protocols, acepromazine (ACP) and acepromazine–methadone (ACP–MET) combination, on tear production measured by the Schirmer Tear Test (STT) 1 on canine eyes were investigated, hypothesizing that both sedation protocols cause a reduction in canine tear production for a variable time. A significant decrease in tear production until 2 h after drug administration compared to basal tear values was observed in the ACP experimental protocol, while in the ACP + MET protocol, this reduction persisted until 8 h. In the ACP + MET group, after 40 min, 100% of dogs showed STT 1 readings lower than 15 mm/min, which could predispose them to corneal injuries. The major reduction in tear production due to the ACP + MET protocol proves the need for adequate corneal hydration, particularly to discourage its use in animals with altered tear production.

**Abstract:**

The purpose of the current study was to investigate the effects of two commonly used sedation protocols in dogs, acepromazine (ACP) and acepromazine–methadone (ACP–MET) combination, on tear production measured by the Schirmer Tear Test (STT) 1. We hypothesized that both sedation protocols cause a reduction in canine tear production for a variable time. Fifteen client-owned dogs were recruited for the study. Each dog was subjected to sedation twice, 2–3 weeks apart, and they were randomly allocated to one of two groups receiving ACP (0.015 mg kg^−1^) or ACP–MET (0.010 mg kg^−1^ and 0.2 mg kg^−1^) intramuscularly. In both eyes, tear production was measured 15 min before sedation (T0) and 20 min (T20 m), 40 min (T40 m), 1 h (T1), 2 h (T2), 4 h (T4) and 8 h (T8), after drug administration. Two-way repeated measures ANOVA, followed by the Bonferroni post hoc test (*p* < 0.05), showed a significant effect of time (*p* < 0.0001) and treatment (*p* < 0.0001). A significant decrease in tear production at T20 m, T40 m, T1 and T2 compared to T0 was observed in the ACP experimental protocol, while in the ACP + MET protocol, this reduction persisted until T8. Comparing the two experimental protocols, no statistically significant differences were observed at T0 or T20 m, and STT 1 values were statistically lower in the ACP + MET than the ACP protocol at the other data points. In the ACP + MET group, at T40 m, 100% of dogs showed STT 1 readings lower than 15 mm/min. This finding is clinically relevant as it can predispose dogs to corneal injuries. The major reduction in tear production due to the ACP + MET protocol proves the need for adequate corneal hydration, particularly to discourage its use in animals with altered tear production. The data obtained provide important information helping clinicians to better manage the drug’s effects on tear production.

## 1. Introduction

In veterinary practice, sedation is commonly used to perform several clinical procedures in order to decrease stress associated with medical examination and diagnostic procedures.

Different sedatives and anesthetic drugs have been reported to cause adverse ocular side effects, such as exposure keratopathy due to loss of eyelid reflex, lagophthalmos, reduced stability of the tear film protecting corneal surface, and decreased basal tear production [1]. Transient dry eye was reported in dogs [2,3,4,5] and cats [6,7]. Perioperative dry eye can result in corneal abrasions due to the loss of corneal integrity. Exposure keratopathy, as a post-anesthesia complication, has been reported in dogs [8,9,10,11], cats [7,12] and horses [13].

Acepromazine and methadone are used in veterinary anaesthesia, alone or in combination, as a tranquilizer or analgesic, respectively, or to reduce the doses of sedatives and anaesthetic drugs [14]; data concerning the effect of their use in sedative protocols on tear production remain inconsistent [4,15,16].

Some authors have reported moderate to marked reductions in tear production in dogs by a combination of phenothiazine agents and full µ-opioid receptor agonists [2,16,17,18].

However, the independent effect of acepromazine on tear production has not been evaluated, and the influence of adding it to protocols containing opioids is not known.

Ocular tear production is commonly measured in ophthalmology using the Schirmer Tear Test 1 (STT 1) devised by Otto Schirmer, a German ophthalmologist, about a century ago. To date, the STT 1 remains the standard test to evaluate ocular tear production in dogs.

In dogs, the premedication before elective general anesthesia with the intramuscular administration of methadone and acepromazine induced a decrease in tear production [18]. Additionally, we have shown that dexmedetomidine reduces canine tear production up to 4 h of more after sedation, making it mandatory to use artificial moisturizing lubricant eye drops to reduce corneal discomfort [5]. In the current study, we expand this earlier study to include an examination of the effects of two more commonly used sedatives in dogs. Therefore, we investigated two different sedation protocols, acepromazine (ACP) and acepromazine–methadone (ACP–MET) combination, and their impact on tear production, measured by STT 1 on canine eye. We hypothesized that both sedation protocols cause a reduction in canine tear production for a variable time.

## 2. Materials and Methods

### 2.1. Animals and Sampling Protocol

Fifteen client-owned dogs, planned to undergo a routine radiologic evaluation, were enrolled in the study. This study was approved by the Department’s Animal Ethics Council (protocol number: 43/2020). All treatments, housing and animal care reported in this study were carried out in accordance with the EU Directive 2010/63/EU for the protection of animals used for scientific purpose. All dogs’ owners provided written informed consent for the study.

Dogs belonging to the Labrador Retriever breed, corresponding to physical status I or II according to the American Society of Anesthesiology (ASA) classification, with baseline STT 1 values ≥ 15 mm/minute, unaffected by systemic or ocular disorders, were included in the study. Exclusion criteria were dogs with an initial baseline STT 1 of less than 10 mm/min and treated with topical or systemic medication.

Dogs underwent complete physical examinations and laboratory exams including complete blood counts and biochemical profiles (urea, creatinine, total protein and albumin).

A complete ophthalmic exam, including biomicroscopy (SL-14 Kowa Slit Lamp), indirect ophthalmoscopy (Heine Omega 120 and Welch Allin Panoptic ophthalmoscopes) and tonometry (Tonopen VET Reichert), was also performed on all patients by a PhD in Veterinary Ophthalmology (S.D.).

Each dog was subjected to sedation twice, 2–3 weeks apart, for diagnostic purposes.

All animals were sedated using two protocols, acepromazine (Prequillan^®^ Fatro S.p.A., Bologna, Italy) (ACP, 0.015 mg kg^−1^) intramuscularly (IM) and acepromazine + methadone (Semfortan Eurovet Animal Health B.V, Bladel, Netherlands) administered separately after 20 min (ACP + MET, 0.010 mg kg^−1^ IM and 0.2 mg kg^−1^ IM, respectively). At the first visit, each dog was randomly (https://www.randomizer.org (accessed on 17 October 2021)) assigned to one of the two different anesthetic protocols and then crossed over to the other at the second anesthetic visit.

The same ophthalmologist, blinded to patient group allocation and order of treatment, performed the ophthalmic examination and STT 1 readings in all dogs at each data point.

The animals were admitted to the hospital at 7:30 a.m. and discharged at 4:30 p.m. Before starting the procedure, all dogs were fasted for 8 h; water was accessible until 30 min before sedation. The time of sedation varied from 20 to 45 min according to the time required for the radiological examination.

During the sedation, the unconscious patients were positioned in sternal recumbency. Heart rate was assessed using a stethoscope, and cardiac rhythm by electrocardiography using lead II; respiratory rate was calculated from excursions of the thorax; a digital thermometer was used for rectal temperature measurement.

**Figure 1:**
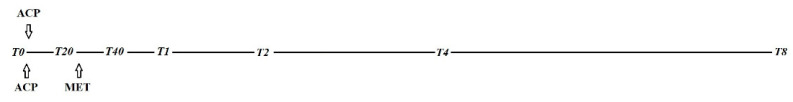
Timeline of events illustrating STT 1 readings, intramuscular administration of acepromazine (ACP, 0.015 mg/kg) alone or acepromazine + methadone after 20 min (ACP + MET, 0.01 mg/kg and 0.2 mg/kg, respectively).

Tear production was measured according to our own methodology previously reported [5]. The STT 1 Tear Test (Tear Strips, Biovision Limited, Dunstable, UK) was performed in both eyes, recording baseline values expressed in millimeters/minute. Tear production was measured 15 min before sedation (T0) and 20 min (T20 m), 40 min (T40 m), 1 h (T1), 2 h (T2), 4 h (T4) and 8 h (T8) after drug administration (Figure 1).

In both treatments, the baseline STT was recorded within 15 min of admission to the hospital, between 7:30 a.m. and 7:45 a.m. The sedation was performed at 8:00 a.m.; successive time points were 8:20 a.m., 8:40 a.m., 9:00 a.m., 10:00 a.m., 12:00 p.m. and 4:00 p.m. All timetables were respected with a variation of no more than 5 min.

After the last STT-1 reading, before the discharge, the corneal integrity was evaluated using slit-lamp biomicroscopy and fluorescein staining of both eyes.

### 2.2. Statistical Analysis

Data obtained were normally distributed (Kolmogorov–Smirnov test). A paired Student’s *t*-test was used to evaluate statistical differences between left and right eyes.

According to Ghaffari et al. [6], data were treated as replicated measures in statistical analysis due to the absence of differences between the two eyes.

To compare tear readings at various data points within (time) and between (treatment) the experimental protocols, two-way repeated measures Analysis of Variance (ANOVA) and the Bonferroni post hoc comparison test were performed; *p* values < 0.05 were considered statistically significant. Results are expressed as mean ± standard deviation (SD). The calculation software Prism 7.0 (Graph Pad Software, San Diego, CA, USA) was used to perform the statistical analysis.

## 3. Results

Enrolled dogs were seven males and eight females of the same breed (Labrador Retriever), with an age of 23.66 ± 12.5 months and body weight of 30.36 ± 3.64 kg (mean ± standard deviation) (Table 1).

Mean values ± SD of basal tear production, expressed in millimeters/min, measured at T0 were 22.01 ± 1.05 in ACP group and 21.66 ± 0.92 in ACP + MET group, respectively.

The application of two-way repeated measures ANOVA showed a significant effect of time (F (6, 348) = 381.1; *p* < 0.0001) and treatment (F (1, 348) = 317.9; *p* < 0.0001).

In particular, a significant decrease in tear production at T20 m, T40 m, T1 and T2 compared to T0 was observed in the ACP experimental protocol, while in the ACP + MET protocol, this reduction persisted until T8.

Both experimental protocols showed the highest percentage of tear production decrease at T40 m, 31.8% and 47.6%, for ACP and ACP + MET, respectively.

In both protocols, a gradual increase in STT1 readings was observed at T1 and T2 with respect to the previous data points (T1 vs. T40 m; T2 vs. T1 and T40 m), although these values remained statistically lower than T0 and T20 m. In the ACP protocol, STT 1 readings reached the basal values at T4 and persisted until T8. They were statistically higher than T20 m, T40 m, T1 and T2 and similar to T0 values. In the ACP + MET protocol, a decrease in STT 1 readings was observed at T20 m and T40 m with respect to the previous data points. Starting from T1 to T8, a gradual increase in STT 1 readings was observed that did not reach the baseline values (Table 2).

Additionally, the comparison between the two experimental protocols showed that no statistically differences were present at T0 and T20 m and that STT 1 values were statistically lower in ACP + MET than ACP protocol at the other data points.

In the ACP group, at T40 m and T1, 35% and 5% of subjects, respectively, showed STT 1 readings lower than 15 mm/min. In ACP + MET group, at T40 m, 100% of dogs showed STT 1 readings lower than 15 mm/min; the percentage of subjects showing this value gradually decreased from T1 to T8 (85%, 45%, 30% and 0%, respectively).

**Figure 2:**
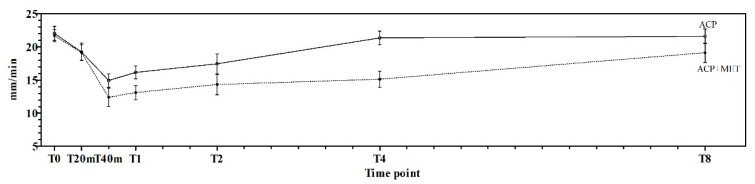
STT 1 readings (mean ± standard deviation), with their statistical differences, at the different data points investigated in the two experimental protocols. ACP indicates the STT 1 values recorded in the group treated with acepromazine. ACP + MET indicates the STT 1 values recorded in the group treated with a combination of acepromazine and methadone.

Figure 2 shows the trend of STT 1 readings at the different data points in the two experimental protocols.

During the experimental period, no dogs exhibited corneal damages.

## 4. Discussion

Many authors have reported that tear production in dogs is affected by tranquilizers, sedatives, opioids and general anesthetic drugs [2,3,4,8,16], although a variable effect on canine tear production was reported due to the inoculation of full μ-opioid receptor agonists, either alone or in association with sedatives or tranquilizers [2,16,17,18].

This study continues previous research on the effect of sedative and anesthetic drugs on tear production in domestic animals, of which a note on the canine tear reduction by dexmedetomidine has already been published [5].

The investigated sedatives are frequently used in canine clinical practice, so we wanted to test them in order to show their possible adverse effects on tear production in dogs.

The results of this study confirmed our hypothesis that acepromazine and methadone sedation protocols could significantly affect canine tear production.

The mechanisms underlying the alteration of tear production due to sedatives and analgesics are numerous with central effect, tear gland vasoconstriction and metabolic alterations, and effective antinociception due to the analgesic action of opioid drugs [2].

Acepromazine causes peripheral vasodilatation, and its cardiovascular effect may explain the slight effect on tear production in this study. In particular, through the postsynaptic inhibition of central dopamine receptors, acepromazine causes depression of the heart rate and blood pressure [19].

The comparison between the two experimental protocols showed that acepromazine affects tear production less in dogs. In contrast to Mouney et al. [4], the tear production after 20 min from the intramuscularly administrated acepromazine differed significantly from baseline readings, even though the STT 1 values did not reach the critical level of less than 15 mm/min, considered a clinical cut-off [18]. In the ACP protocol, this condition was observed only in seven subjects 40 min after the administration of this drug.

In the ACP + MET protocol, a strong decrease in tear production was recorded after 40 min and 1 h post-sedation, persisting in five animals until four hours post-sedation.

The association of a sedative drug with a full µ-opioid agonist resulted in a marked decrease in tear production, suggesting an additional effect in reducing the STT 1 readings, as previously observed by Volk et al. [18], who tested the combination of the two drugs administered at the same time in the same syringe. The simultaneous inoculation of methadone may maintain the down regulation of tear production by acepromazine, due to its sedative effect that can persist until 4–6 h. Some authors reported that the synergism between acepromazine and methadone provides more profound sedation in dogs than either drug administered individually, with an early onset of action and a longer effect [14]. Methadone alone or in combination also provides a long-lasting antinociceptive effect [20]. Moreover, some authors reported that in feline patients, the excitation, by increasing sympathetic tone to the lacrimal gland, did not influence STT values [21]. Therefore, the major inhibitory effect on canine tear production observed in this study by acepromazine and methadone combination may be due to the analgesic action of the opioid agent. Opioids have been showed to affect mu, delta and kappa receptors, leading to the regulation of many physiological responses. In particular, their coupling with G protein receptors impacts parasympathetic function including not only gastrointestinal motility and salivary secretion, but also playing a critical role in modulating tear production [22,23].

In the evaluation for a long period of STT-1 modification after sedation, the circadian rhythmicity of canine tear production should be taken in consideration. Throughout the day, tear production increases by 0.7 mm/min during the photophase [24] and reaches the acrophase during the night in normal dogs [25].

In clinical practice, an opioid is often added to a protocol due to synergistic effect with the sedative. Additionally, opioids are required for appropriate multimodal analgesia for invasive/painful procedures. The major reduction in tear production due to the ACP + MET protocol proves the need for a supplementary treatment to support corneal hydration in this timeframe. This finding is relevant to clinicians when dealing with animals that need sedation, particularly when the protocol is used in animals with altered tear production.

The finding of STT 1 values below 15 mm/min, especially in ACP + MET protocol, is clinically relevant as they can predispose to corneal injuries.

## 5. Conclusions

The data obtained in the present research provide important information, helping clinicians to better manage the effect of sedative drugs on tear production. Sedation may be required for several clinical procedures in animals affected by tear production alterations and other eye diseases. Therefore, the use of ocular ointment in animals sedated by ACP + MET may be particularly important post-procedure to protect the cornea.

## Figures and Tables

**Table 1:** Signalment of enrolled dogs.

Dogs	Gender	Age (Months)	Body Weight (kg)	Drug Received at First Visit
1	M	12	25	ACP
3	M	48	35	ACP
6	M	24	31.5	ACP
8	F	12	35	ACP
10	F	48	32.5	ACP
14	F	20	30	ACP
15	F	22	29	ACP
2	F	36	29.5	ACP + MET
4	F	12	26	ACP + MET
5	F	12	33.5	ACP + MET
7	M	36	28	ACP + MET
9	M	24	33	ACP + MET
11	M	18	27	ACP + MET
12	M	16	34	ACP + MET
13	F	15	32	ACP + MET
MEAN ± SD		23.66 ± 12.57	30.36 ± 3.64	

**Table 2:** Mean values ± standard deviation of STT 1 readings, expressed in mm/min, in the different experimental protocols (acepromazine: ACP; acepromazine + methadone: ACP + MET) in all data points. Their statistical differences were also reported: a vs. T0 *p* < 0.001; b vs. T20 *p* < 0.001; c vs. T40 *p* < 0.001; d vs. T1 *p* < 0.001; e vs. T2 *p* < 0.05; f vs. T4 *p* < 0.001; * vs. ACP *p* < 0.001.

	Data Points						
Experimental Protocol	T0	T20	T40	T1	T2	T4	T8
ACP	22.01 ± 1.05	19.23 ± 1.32	14.91 ± 1.00	16.15 ± 0.96	17.43 ± 1.50	21.35 ± 1.02	21.58 ± 1.11
Time effect		a	ab	abc	abcd	bcde	bcde
ACP + MET	21.66 ± 0.92	19.11 ± 1.15	12.40 ± 1.37 *	13.10±1.07 *	14.31±1.52 *	15.11 ± 1.23 *	19.10 ± 1.47 *
Time effect		a	ab	abc	abcd	abcde	acdef

## Data Availability

The data presented in this study are available on request from the corresponding author.
